# Influence of surface treatment and cyclic loading on the durability of repaired all-ceramic crowns

**DOI:** 10.1590/S1678-77572010000200015

**Published:** 2010

**Authors:** Ahmed ATTIA

**Affiliations:** 1 MScD, Dr Med Dent, PhD, Associate Professor, Department of Conservative Dentistry and Fixed Prosthodontics, Faculty of Dentistry, Mansoura University, Egypt.

**Keywords:** All-ceramic crown, Repair techniques, Fracture load

## Abstract

**Objective:**

This study investigated the durability of repaired all-ceramic crowns after cyclic loading.

**Material and methods:**

Eighty In-ceram zirconia crowns were fabricated to restore prepared maxillary premolars. Resin cement was used for cementation of crowns. Palatal cusps were removed to simulate fracture of veneering porcelain and divided into 4 groups (n = 20). Fracture site was treated before repair as follows: roughening with diamond bur, (DB); air abrasion using 50 µm Al_2_O_3_, (AA) and silica coating using Cojet system followed by silane application, (SC). Control group (CG) 20 specimens were left without fracture. Palatal cusps were repaired using composite resin. Specimens were stored in water bath at 37°C for one week. Ten specimens of each group were subjected to cyclic loading. Fracture load (N) was recorded for each specimen using a universal testing machine. Two-way analysis of variance (ANOVA) and Tukey honestly significant difference (HSD) test (α=.05) were used for statistical analysis.

**Results:**

There was statistically significant difference between control and tested groups, (p<0.001). Post Hoc analysis with the Tukey HSD test showed that cyclic loading fatigue significantly decreased means fracture load of control and test groups as follows (CG, 950.4±62.6 / 872.3±87.4, P = 0.0004), (DB, 624.2 ±38 / 425.5± 31.7, P <.001), (AA, 711.5 ±15.5 / 490 ± 25.2, p <0.001) and (SC, 788.7 ± 18.1 / 610.2 ± 25.2, P <.001), while silica coating and silane application significantly increased fracture load of repaired crowns (p<0.05).

**Conclusion:**

Repair of fractured Inceram zirconia crowns after chairside treatment of the fracture site by silica coating and silane application could improve longevity of repaired In-ceram zirconia crowns.

## INTRODUCTION

In spite of the advantages of all-ceramic restorations including life-like appearance, biocompatibility and durability, there are still disadvantages to their use clinically^2^. Fracture of veneering ceramics still remains the primary cause of failure of all-ceramic crowns18. Core- veneer interface is one of the weakest aspects of layered all-ceramic crowns1, so that ceramic chipping or cracking are possible^1^. Fracture of ceramic restorations lead to increased cost, discomfort, time and labor when a replacement is required^24^. Because it is arduous to remove these restorations from the mouth, ceramic restorations may be repaired intraorally^3^. Intraoral repair may help in lengthening the life span of the fractured restoration^19,27^. A number of ceramic repair materials and techniques are available for treating this dilemma^11,16,20^.

Different ceramic surface treatments have been introduced to improve resin bonding to ceramics^4,5,8,12,23,25,26^. Opposite to silica-based ceramics, alumina and zirconia based ceramics are inert to conventional hydrofluoric acid etching^6,10^ so other surface treatments such as silica coating are used to improve resin bonding to these ceramics^17^. Moreover, repair of fractured ceramic restoration with silica-coating and silane application is a relevant adhesion promotion method^13,21^. Clinically, restorations are subjected to masticatory forces under dry and wet corrosive conditions^14,15^. *In vitro* studies should replicate the clinical conditions^7^,and thus various methods have been developed to simulate oral environment such as cyclic loading fatigue^7,9^. Many factors could influence the durability of repaired all-ceramic crowns *in vitro*. These factors are storage conditions, cyclic loading and surface treatments of the fractured restorations before repair^12,19^. The oral environmental conditions, direction and magnitude of acting forces and time strongly influenced the longevity of dental restorations in term of wear and degradation^11^.

The purpose of this *in vitro* study was to evaluate the durability of direct repair of fractured In-ceram zirconia crowns after different surface treatment and cyclic loading. The tested hypothesis was that silica coating of the fracture site using Cojet system followed by silane application increase fracture load of the repaired crowns compared to the other methods of surface treatments of the fracture site before repair.

## MATERIAL AND MEHTODS

Eighty carious and crack-free human maxillary premolars were prepared according to the following standardized preparation criteria^2,7^: 6degree axial taper, 1 mm shoulder finish line placed 0.5 mm occlusal to the cementoenamel junction (CEJ), 2 mm occlusal reduction and occluso-gingival height of 5 mm. Prepared premolars were fixed in metal rings. First the root portion, 2 mm away from the CEJ, was coated with an artificial periodontal membrane made from a gum resin^2,7^ (Anti-Rutsch-Lack, Wenko-Wenselaar, Hilden, Germany). Each specimen was coronally covered with wax (Modeling Wax; Cavex, Haarlem, Holland) and then the root was dipped once into the gum resin. After the gum resin had dried, the excess at the root tip was removed with a scalpel so that an almost 0.2 mm uniform coating covered the root surface^2,7^. This coating allowed tooth mobility similar to physiological mobility of the natural teeth^7^. Then the specimens were fixed in 15 mmdiameter metal rings using fast setting polyester resin (Technovit 4000; Heraeus-Kulzer, Wehrheim, Germany). A one-stage impression technique using putty and light bodied vinyl polysiloxane material (President, Coltène Whaledent, Altstatten, Switzerland) was used for making an impression of the prepared tooth. Stone (GC Fujirock EP, GC Belgium N.V, Leuven, Belgium) definitive dies were prepared from these impressions. After duplication, Vita In-ceram sprint special plaster (Vita, Bad Sackingen, Germany) was used for pouring the duplication to produce special plaster dies.

In-ceram zirconia (Vita, Bad Sackingen, Germany) powder was mixed and built up on the special plaster dies using acrylic brush according to the manufacturer instructions to build up In-ceram zirconia cores. Each core was veneered with conventional powder slurry porcelain (VITA VM7, Vita, Bad Sackingen) to obtain anatomy of maxillary premolar. A custom made silicone mold fabricated according to the dimensions of the maxillary right second premolar removed from a dentiform (# 0623321; KaVo, Biberach, Germany) was used to standardize final dimensions of crowns, 1.5 mm thickness at the axial walls and 2 mm thickness at the occlusal surface^2,7^. A caliper (Mestra, Bilbao, Spain) with a measuring accuracy of 0.1 mm was used to confirm the intercuspal distance from buccal cusp tip to the palatal cusp tip to be 3 mm. Also, the ceramic thickness at the central occlusal fissure, slopes and cusp tips of both buccal and palatal cusps were determined to be within 2.0 ± 0.1 mm using the calipers^2,7^.

**Figure 1:**
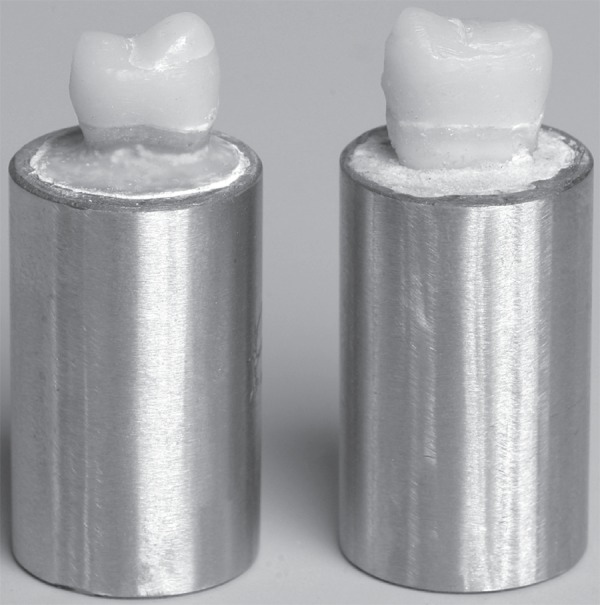
Fractured and repaired specimens

**Figure 2:**
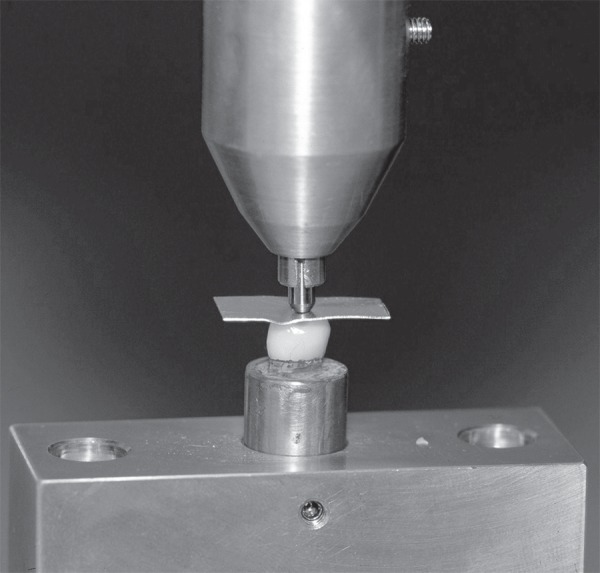
Applying the compressive load along the long axis of test and control specimens

**Figure 3:**
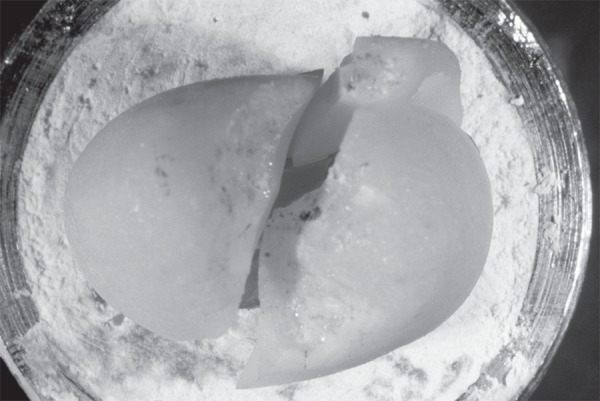
Fractured specimen after application of compressive load

The intaglio surfaces of the crowns were treated with airborne particle abrasion using 50 µm aluminum oxide particles, followed by ultrasonic cleaning in distilled waMeans, standard deviations, minimum Vivadent, Schaan, Liechtenstein) was applied to the intaglio surfaces of the crowns and left 2 minutes to dry. Multilink Primer A+B (1:1) (Ivoclar Vivadent) were mixed and applied evenly to the adherent tooth surface and dried with oil free air. Equal amounts of adhesive resin cement (Multilink automix, Ivoclar Vivadent) were extruded, mixed for 20 s and applied to the intaglio surfaces of crowns. Each crown was seated to its respective prepared tooth and kept under a static pressure of 40 N for 7 minutes in a loading apparatus^7,14^. The excess of luting cement at the margin was removed immediately. One hour after cementation, veneering porcelain of palatal cusps of sixty crowns were completely removed using porcelain finishing stone (# 6844.374-016, Komet Medical, Germany) to expose the In-ceram zirconia core to simulate fracture of veneering porcelain with core exposure^11^ (Figure 1). The other 20 specimens left without palatal cusp fracture served as control group. Fractured specimens were divided into 3 groups (n=20) according to surface treatment of the frature site before repair as follow; roughening with diamond bur, (DB), air borne particle abrasion with 50 µm Al_2_O_3_, at a pressure of 2.8 bars for 5 s at a distance of 10 mm (AA) and silica coating using Cojet system (3M ESPE, Seefeld, Germany) as follow: air borne particle abrasion with 30-µm SiO_2_ particles at a pressure of 2.8 bars for 20 s at a distance of 10 mm followed by silane application^28^ (ESPE Sil, 3M ESPE), (SC). Five minutes were allowed to elapse for silane reaction^28^. Bonding agent (Visio Bond, 3M ESPE) was applied on the fracture site of all test groups to enhance bonding at ceramic/composite interface and light cured for 20 s at 5 mm distance (FutoLux 2, Carlo De Gorgi, Baranzate di Bollate, Italy). Light activated, radiopaque, restorative composite (Filtek Z250, 3M ESPE) was applied in layers and adapted to the fracture site using plastic instrument and light cured for 40 s at 5 mm distance (FutoLux 2, Carlo De Gorgi) to build up the palatal cusp. The same caliper was used again to confirm the intercuspal distance from buccal cusp tip to the palatal cusp tip to be 3 mm. Composite finishing stone (1112F, 3118F and 3195FF, KG Sorensen, Barueri, SP, Brazil) was used to remove excess composite and to achieve the desired contour of the repaired cusp. The cusp was then polished using polishing disc (Soflex, 3M ESPE). One hour after repair test and control groups were stored in water bath at 37°C for 1 week. To mimic the intraoral conditions, half of the specimens in each group (n = 10) were fatigued in a computerized masticatory simulator^7^ (Willitec, Munich, Germany) under wet conditions for 250,000 masticatory cycles. The loading cycle frequency was 1.2 Hz, with a kinetic energy of 2,250 ×10^6^ J, maximum load of 49 N and minimum load of 0 N and lateral component 0.3 mm. Steatite ceramic balls (4 mm diameter; Hoechst Ceram Tec, Wunsiedel, Germany) were used as antagonistic surfaces to simulate the opposite teeth. Specimens were mounted on stubs using auto-polymerizing resin (Vitron M; 3M ESPE) and were then fixed to the upper specimen holders in the masticatory simulator. The position of each test specimen was adjusted to assure that the opposing ceramic ball contacted the triangular ridge of the palatal cusp of the crown^2,7^. The other half (n = 10) of each subgroup was fractured without fatigue. For determination of the fracture load, a stainless steel bar with a 4-mm diameter ball end-mounted in universal testing machine (Type 500, Lloyd Instrument, England) was used to apply compressive load along the long axis of test and control specimens at a crosshead speed of 1 mm/min until fracture^2,7^. The compressive load (N) was centered on the midline fissure of each crown. To avoid uneven distribution of the applied force and avoid loading stress peaks on repaired cusp, a 1 mm thin piece of polyethylene vacuum-forming foil (copyplast 1.0, Scheu-Dental, Iserlohn, Germany) was placed between the stainless steel bar and the crown (Figure 2). The compressive load required to cause fracture (N) was recorded for each specimen (Figure 3). Two-way analysis of variance (ANOVA) followed by serial one-way ANOVA with and without cyclic and Tukey’s HSD test at significance level of .05 were used for statistical analysis of the data.

## RESULTS

Means, standard deviations, minimum and maximum fracture loads of control and test groups without and with cyclic loading are listed in Table 1. Each factor, either surface treatment or cyclic loading fatigue, significantly influencedthe fracture load of control and test groups (p<0.001). Also the interaction of the 2 factors (surface treatment x cyclic loading fatigue) significantly influenced fracture load of all groups (p<0.001).

**Table 1:** Means ± standard deviations (SD), minimum and maximum fracture load and p value of control and test groups without and with cyclic loading in N

**Groups**	**Without cyclic loading**		**With cyclic loading**		**p-values**
	**Mean ± SD**	**Minimum -** ** Maximum**	**Mean ± SD**	**Minimum -** ** Maximum**	**Cyclic loaded** ** vs unloaded**
Roughening with diamond bur (DB)	624.2 ±38	580 - 703	425.5± 31.7	380 -480	* p <0.001
Air abrasion using 50 pm Al2O3 (AA)	711.5 ±15.5	689 - 740	490 ± 25.2	468 -520	* p <0.001
Silica coating using Cojet system (SC)	788.7 ± 18.1	760 -811	610.2 ± 25.2	573 - 643	* p <0.001
Control Group (CG)	950.4±62.6	846 -1012	872.3±87.4	740 - 990	* p =0.0004

Tukey test at p =0.05)

* Statistically significant difference (α = .05).

There was statistically significant difference between repaired groups after different surface treatments (p<0.001) either with or without cyclic loading.

Post-hoc analysis with the Tukey HSD test showed that cyclic loading fatigue (Table 1) significantly decreased mean fracture loads of control and test groups as follow (CG, 950.4±62.6 / 872.3±87.4, p = 0.0004), (DB, 624.2 ±38 / 425.5± 31.7, p<0.001), (AA, 711.5 ±15.5 / 490 ± 25.2, p<0.001) and (SC, 788.7 ± 18.1 / 610.2 ± 25.2, p<0.001).

Considering different surface treatments without cyclic loading mean fracture loads of control group (CG) was significantly higher than mean fracture loads of all test groups (DB, p < 0.001), (AA, p<0.001) and (SC, p<0.001). Also mean fracture loads of (SC) group was significantly higher than mean fracture loads of other test groups as follow (DB, p<0.001) and (AA, P = 0.005) and mean fracture loads of group (AA) was significantly higher than mean fracture loads of group (DB, p<0.001).

With cyclic loading mean fracture loads of control group (CG) was significantly higher than mean fracture loads of all test groups as follow (DB, p<0.001); (AA, p<0.001) and (SC, P<0.001). Also mean fracture loads of group (SC) was significantly higher than mean fracture loads of other test groups as follow (DB, p<0.001) and (AA, p<0.001) and mean fracture loads of group (AA) was significantly higher than mean fracture loads of group (DB, P = 0.034).

## DISCUSSION

The dynamic nature of the stresses due to mastication promotes crack growth. In addition, corrosive components of the oral environment enhance the growth of microcracks and consequently porcelain fracture^15^. Both *in vivo* and *in vitro* studies evaluated the durability of different porcelain repair materials and techniques^11,12^. However, *in vitro* studies were focused on tensile, shear and flexural strength tests in Mpa^11^. This study was not a test of bond strength but rather an evaluation of the durability of the repaired all-ceramic crown as a single unit after cyclic loading fatigue. It was reported that humans have an average of 250,000 masticatory cycles per year^9^. Therefore in this study, instead of bond strength in Mpa, fracture loads in N of repaired and control specimens were recorded after cyclic loading fatigue for 250,000 cycles to replicate the clinical conditions for one year^9^. However using of water instead of artificial saliva plus the limited numbers of specimens used are limitations of this *in vitro* study.

Ozcan and Niedermeier^25^ reported that failures of intraoral repair using composite resin were due to trauma, masticatory forces or improper bonding procedures. However Creugers, Snoek and Kayser^11^ in their clinical study attributed failure of occlusal repair of metal-ceramic crowns to surface deterioration of the composite resin used but not for the repair systems. In agreement with both in vivo^11,25^ and *in vitro* literatures^7,9,20^ in this study cyclic loading fatigue significantly decreased fracture load of test and control groups. Fatigue is described as phenomenon in which the characteristics of materials change over time under constant conditions^7^. All-ceramic crowns are process dependent brittle materials and have little capacity to deform and, thereby, decrease the concentration of stresses at a crack tip^7,18^. Cyclic loading, especially under wet conditions, results in the propagation of small cracks which might initiate from processingrelated porosities within the crowns^7,14,18^. These cracks fuse to a growing fissure that weakens the crowns^7,18^.

Another factor that may decrease the fracture load of test and control groups was the static fatigue, a stress dependent chemical reaction between the water and surface flaws, which caused the flaw to grow to a critical dimension, which then allowed for spontaneous crack propagation^7,14^. The combined negative effect of cyclic loading and the wet environment caused control and repaired crowns to fracture under relatively low compressive load as reported in other studies^7,14,18^.

Moreover repaired all-ceramic crowns are complex restoration where composite resin which is less stiff material is bonded to stiff material (In-ceram zirconia) in the fracture site. In this complex restorative system, when load was applied, high tensile stresses developed in the ceramic/composite interface directly below the loaded area^7,18^. These interfacial stresses are highly sensitive to variations in the elastic modulus of the materials and developed due to strain differences of the ceramic and composite resin^7,18^.

In-ceram zirconia is non silica based ceramics so it is not responding to etching using hydrofluoric acid ^6,17^. Moreover hydrofluoric acid is poisonous, caustic, foaming liquid that is extremely irritable to skin and lungs^12^. Therefore during intraoral repair of fractured all-ceramic crowns omitting the step of HF acid etching might be an interesting step^12^. In this study fractured crowns were treated using one of the following three methods, roughening with diamond bur, airborne particle abrasion or silica coating using Cojet system. Silane coupling agent was applied after silica coating to achieve a durable chemical bond of composite resin to the repaired specimens as reported by several literatures^6,17,21^. Direct composite resin was used for building up the fractured palatal cusps because composite resin was the material of choice for intraoral porcelain repair for several years^11^. Silica coating followed by silane application significantly increased fracture load of repaired specimens compared to the other surface treatments with and without cyclic loading. Amaral, et al.^4^ reported that conditioning with silica coating and silanization improved bond strengths to In-ceram zirconia than with airborne particle abrasion only. Moreover Frankenberger, Kramer and Sindel^13^, Matinlinna and Vallittu^21^ reported that silica coating followed by silane application is a suitable treatment for the intraoral repair using composite resin. The higher fracture load of repaired specimens after silica coating could be attributed to the creation of topographic pattern allowing for micromechanical bonding plus chemical bonding because of using silane coupling agent at composite resin/silica coated ceramic surface^23,26^. In this case the repaired specimens reacted against the applied dynamic and static loads as single unit, consequently increased the fracture load.

Bond strength at ceramic/composite interface in groups repaired after roughening with diamond bur or airborne particle abrasion using Al_2_O_3_ was only micromechanical bonding due to flow of bonding agent into the micromechanical pores caused by diamond bur or airborne particle abrasion using alumina particles^26^. However, surface roughening using diamond burs could increase crack initiation and propagation through already weakened fractured ceramic surface which could result in failure of repair^26^. The cumulative negative effect of roughening with diamond bur, cyclic loading fatigue and hydrolytic effect of water on the adhesive bond at ceramic/ composite interface significantly decreased fracture load of this group compared to other test groups.

## CONCLUSIONS

Within the limitations of this *in vitro* study, the following conclusions may be drawn: 1. Cyclic loading fatigue significantly decreased mean fracture loads of all groups; 2. Mean fracture loads of repaired groups after silica coating and silane application was significantly higher than the other two groups with and without cyclic loading; 3. Repair of the fractured In-ceram zirconia crowns after surface treatment with silica coating using Cojet system and silane application could be an alternative technique for improving durability of intraoral repair using composite resin.

## References

[1] Aboushelib MN, De Jager N, Pallav P, Feilzer AJ (2005). Microtensile bond strength of different components of core veneered
all-ceramic restorations. Dent Mater.

[2] Attia A, Kern M (2004). Fracture strength of all-ceramic crowns luted using two bonding
methods. J Prosthet Dent.

[3] Appeldoorn RE, Wilwerding TM, Barkmeier WW (1993). Bond strength of composite resin to porcelain with newer generation
porcelain repair systems. J Prosthet Dent.

[4] Amaral R, Ozcan M, Bottino MA, Valandro LF (2006). Microtensile bond strength of a resin cement to glass infilterated
zirconia-reinforced ceramic: The effect of surface conditioning. Dent Mater.

[5] Amaral R, Ozcan M, Valandro LF, Balducci I, Bottino MA (2008). Effect of conditioning methods on the microtensile bond strength of
phosphate monomer-based cement on zirconia ceramic in dry and aged
conditions. J Biomed Mater Res B Appl Biomater.

[6] Atsu SS, Kilicarslan MA, Kucukesmen HC, Aka PS (2006). Effect of zirconium-oxide ceramic surface treatments on the bond
strength to adhesive resin. J Prosthet Dent.

[7] Attia A, Kern M (2004). Influence of cyclic loading and luting agents on the fracture load of
two all-ceramic crown systems. J Prosthet Dent.

[8] Bottino MA, Valandro LF, Scotti R, Buso L (2005). Effect of surface treatments on the resin bond to zirconium-based
ceramic. Int J Prosthodont.

[9] Att W, Kurun S, Gerds T, Strub JR (2006). Fracture resistance of singletooth implant-supported all-ceramic
restorations: an in vitro study. J Prosthet Dent.

[10] Borges GA, Sophr AM, De Goes MF, Sobrinho LC, Chan DC (2003). Effect of etching and airborne particle abrasion on microstructure of
different dental ceramics. J Prosthet Dent.

[11] Creugers NHJ, Snoek PA, Kayser AF (1992). An experimental porcelain repair system evaluated under controlled
clinical conditions. J Prosthet Dent.

[12] Fan PL (1991). Council on dental materials instruments and equipments. Porcelain
repair materials. J Am Dent Assoc.

[13] Frankenberger R, Kramer N, Sindel J (2000). Repair strength of etched vs silica-coated metal-ceramic and
all-ceramic restorations. Oper Dent.

[14] Correr L, Cattell MJ, Glover RH, Knowles JC (1998). Investigation of the dry and wet fatigue properties of three
allceramic crown systems. Int J Prosthodont.

[15] Herrmann M, Rottenegger R, Tinschert J, Marx R (1992). The effect of corrosive environment on the porcelain-to-metal bond-a
fracture mechanics investigation. Dent Mater.

[16] Haselton DR, Diaz-Arnold AM, Dunne JT Jr (2001). Shear bond strengths of 2 intraoral porcelain repair systems to
porcelain or metal substrates. J Prosthet Dent.

[17] Heikkinen TT, Lassila LV, Matinlinna JP, Vallittu PK (2007). Effect of operating air pressure on tribochemical
silica-coating. Acta Odontol Scand.

[18] Kelly JR (1999). Clinically relevant approach to failure testing of allceramic
restorations. J Prosthet Dent.

[19] Kumbuloglu O, User A, Toksavul S, Vallittu PK (2003). Intra-oral adhesive systems for ceramic repairs: a
comparison. Acta Odontol Scand.

[20] Leibrock A, Degenhart M, Behr M, Rosentritt M, Handel G (1999). In vitro study of the effect of thermo- and load-cycling on the bond
strength of porcelain repair systems. J Oral Rehabil.

[21] Matinlinna JP, Vallittu PK (2007). Bonding of resin composites to etchable ceramic surfaces - an insight
review of the chemical aspects on surface conditioning. J Oral Rehabil.

[22] Ozan M, Van Der Sleen JM, Kurunmaki H, Vallittu PK (2006). Comparison of repair methods for ceramic fused to metal
crowns. J Prosthodont.

[23] Ozcan M, Vallittu PK (2003). Effect of surface conditioning methods on the bond strength of luting
cement to ceramics. Dent Mater.

[24] Ozcan M (2002). The use of chairside silica coating for different dental applications:
a clinical report. J Prosthet Dent.

[25] Ozcan M, Niedermeier W (2002). Clinical study on the reasons for and location of failures of
metal-ceramic restorations and survival of repairs. Int J Prosthodont.

[26] Ozcan M (2003). Evaluation of alternative intra-oral repair techniques for fractured
ceramic- fused- to -metal restorations. J Oral Rehabil.

[27] Rosentritt M, Behr M, Kolbeck C, Lang R, Handel G (2000). In vitro repair of all-ceramic and fibre-reinforced composite
crowns. Eur J Prosthodont Restor Dent.

[28] Valandro LF, Ozcan M, Amaral R, Leite FPP, Bottino MA (2007). Microtensile bond strength of a resin cement to silica-coated and
silanized In-Ceram Zirconia before and after aging. Int J Prosthodont.

